# Synergistic Integration of Local and Global Information for Critical Edge Identification

**DOI:** 10.3390/e26110933

**Published:** 2024-10-31

**Authors:** Na Zhao, Ting Luo, Hao Wang, Shuang-Ping Yang, Ni-Fei Xiong, Ming Jing, Jian Wang

**Affiliations:** 1 Key Laboratory in Software Engineering of Yunnan Province, Yunnan University, Kunming 650091, China; zhaonayx@126.com (N.Z.); 18979213005@163.com (T.L.); wangh991015@gmail.com (H.W.); yangshuangping@mail.ynu.edu.cn (S.-P.Y.); xiongnifei@stu.ynu.edu.cn (N.-F.X.); 2 Big Data Research Center, University of Electronic Science and Technology of China, Chengdu 610056, China; 3 School of Artificial Intelligence & Information Engineering, West Yunnan University, Lincang 677000, China; proofle@163.com; 4 College of Information Engineering and Automation, Kunming University of Science and Technology, Kunming 650504, China

**Keywords:** network science, complex networks, critical edge identification, edge percolation, robustness

## Abstract

Identifying critical edges in complex networks is a fundamental challenge in the study of complex networks. Traditional approaches tend to rely solely on either global information or local information. However, this dependence on a single information source fails to capture the multi-layered complexity of critical edges, often resulting in incomplete or inaccurate identification. Therefore, it is essential to develop a method that integrates multiple sources of information to enhance critical edge identification and provide a deeper understanding and optimization of the structure and function of complex networks. In this paper, we introduce a Global–Local Hybrid Centrality method which integrates a second-order neighborhood index, a first-order neighborhood index, and an edge betweenness index, thus combining both local and global perspectives. We further employ the edge percolation process to evaluate the significance of edges in maintaining network connectivity. Experimental results on various real-world complex network datasets demonstrate that the proposed method significantly improves the accuracy of critical edge identification, providing theoretical and methodological support for the analysis and optimization of complex networks.

## 1. Introduction

Complex networks are defined by properties such as self-organization, scale-freeness, and small-world phenomena. Many real-world systems can be abstracted and modeled using complex network theory, including social networks [1,2], transportation systems [3,4] power grids [5,6], and biological systems [7]. These systems are typically represented as networks of nodes and edges, where nodes represent entities in the system, and edges represent the interactions between these entities.

While numerous approaches to ranking the importance of nodes have been proposed, the identification of critical edges has been comparatively overlooked, despite its importance in practical applications. In complex networks, critical edges refer to connections that significantly impact the functionality and stability of the network. The removal of these edges can severely disrupt the overall connectivity and structural stability of the network, potentially leading to its collapse. Therefore, accurately identifying critical edges is essential for maintaining the robustness of the network. Optimal percolation theory [8] provides an important theoretical foundation for the identification of critical edges. This theory originates from physics and was initially used to study the permeation behavior of fluids in porous media. In the context of complex network research, percolation theory is widely applied to analyzing the robustness and connectivity of networks. Specifically, percolation refers to the phenomenon where the network structure gradually disintegrates during the incremental removal of nodes or edges. Optimal percolation emphasizes selectively removing nodes or edges according to specific strategies or planning the removal sequence of nodes or edges in a particular order, thereby causing the primary connected component of the network to collapse rapidly and ultimately split into several small, disconnected subcomponents. Within the framework of optimal percolation theory, critical edges are defined as edges that are prioritized for removal during the disconnection process. This prioritization is due to the fact that the removal of these edges can maximally accelerate the disintegration of the primary connected component within the network, leading to its swift collapse. Thus, an accurate critical edge identification method should be able to quantify the extent of network damage caused by the removal of a single edge during the percolation process, thereby formulating effective edge removal strategies aimed at inducing the network’s collapse or disintegration as quickly as possible, achieving optimal percolation.

Several algorithms have been developed to identify critical edges. Girvan [9] et al. introduced the concept of edge betweenness, suggesting that an edge’s importance increases with the number of shortest paths passing through it. Holme [10] et al. suggested that the product of the degrees of the nodes at both ends of an edge is a key measure of edge importance. Liu [11] et al. introduced a method for calculating the propagation strength of edges. Onnela [12] et al. identified critical edges from the perspective of structural and information flow control, introducing the topological overlap method. Cheng [13] et al. proposed the bridging coefficient, viewing edges as bridges connecting two factions. Zhao [14] et al. developed the second-order neighbor index, utilizing higher-order common neighbor information to identify critical edges. Yu et al. [15] introduced the BCCMOD algorithm, which integrates edge betweenness centrality and the clique structure within the network to assess the significance of edges. Chen et al. [16] proposed an edge importance ranking method based on improved structural holes and information entropy, which effectively enhances the accuracy of identifying critical edges by taking into account both the network structure and transmission performance. Additionally, there are other critical edge identification algorithms based on vector features [17,18] and information dissemination theories [19,20].

Despite these advancements, most of the existing methods depend heavily on either global or local information, which limits their ability to comprehensively capture the multi-layered characteristics and complexity of critical edges within networks [21]. On the one hand, local methods, such as second-order neighborhood (SN), perform exceptionally well in networks with prominent community structures. This is because local methods are well-suited to capture close connections within community structures. Additionally, local methods have lower computational complexity, making them suitable for large-scale networks that require fast identification of critical edges. However, these methods have limitations in fully capturing cross-community edges and global network characteristics, often overlooking edges that are important at the cross-community or global level. In networks with strong inter-community connectivity, multi-level community structures, or dense networks without obvious community boundaries, this local perspective struggles to effectively identify critical edges between communities. On the other hand, global methods, such as edge betweenness (EB), approach from a global perspective and are better suited to identifying cross-community edges. However, they also have certain drawbacks. These methods are less sensitive to local details, which can result in the neglect of locally critical edges. Additionally, global methods are more susceptible to noise and outliers in the network, leading to reduced accuracy. Therefore, in practical applications, global methods may find it difficult to accurately distinguish truly critical edges.

To address these shortcomings, this paper introduces a Global–Local Hybrid Centrality (GLHC) method that integrates global and local information to provide a more comprehensive evaluation of the edge significance in networks. The core idea of GLHC is to combine both global and local network features to assess edge importance. For local features, GLHC adopts the second-order neighborhood index, which has shown effective performance in measuring importance, and incorporates the first-order neighbor index to compensate for the shortcomings of the second-order neighborhood index in sparse networks. For global features, GLHC introduces edge betweenness as a measure to mitigate the limitations of relying solely on local information, which may overlook the broader role of edges within the entire network. To validate the performance of GLHC, we conducted a series of experiments, including robustness, connectivity, and monotonicity tests, on several real-world unweighted networks. The experimental results demonstrate that the GLHC method consistently outperforms existing approaches. Specifically, the advantages of the GLHC method include the following:

(1) By combining local information with global information, the GLHC method provides a more comprehensive assessment of edge importance. Local information helps capture fine-grained structures within communities, while global information offers a macroscopic view of cross-community connections. This combination enables the GLHC method to evaluate edge importance at multiple levels, resulting in more thorough and accurate identification of critical edges.
(2) The GLHC method shows strong stability in response to changes and disturbances in the network topology, ensuring network connectivity and the overall performance.

## 2. The Proposed Method

This section introduces the critical edge identification methods used in the experiments. Section 2.1 presents the existing critical edge identification methods, which serve as baseline methods for evaluating the performance of GLHC. Section 2.2 introduces the proposed GLHC method.

### 2.1. Baseline Methods

#### 2.1.1. Degree Product (DP)

Degree Product (DP) [10] is the simplest metric for critical edge measurement. It assesses the importance of an edge by multiplying the degrees of the two nodes it connects, defined as follows:

(1) $$DP(e_{ij})=k_{i}\times k_{j}$$

where the edge $e_{ij}$ connects node i and node j, and $k_{i}$ and $k_{j}$ represent the degrees of node i and node j, respectively.

#### 2.1.2. Edge Betweenness (EB)

Edge betweenness (EB) [9] is calculated based on the shortest paths in the graph. For an undirected network, edge betweenness is defined as the proportion of all shortest paths that pass through a particular edge.

(2) $$EB(e_{ij})=\sum_{s\neq t}\frac{n(s,t,e_{ij})}{n(s,t)}$$

where n(s,t) is the total number of shortest paths from node s to node t, and $n(s,t,e_{ij})$ is the number of those shortest paths that pass through edge $e_{ij}$. The edge betweenness of an edge is the sum of $n(s,t,e_{ij})$ over all pairs of nodes, divided by the sum of n(s,t).

#### 2.1.3. Bridgeness (BN)

Bridgeness (BN) [13] focuses on the role of an edge as a bridge between different communities. It is defined as

(3) $$BN(e_{ij})=\frac{\sqrt{S_{i\;}S_{j}}}{S(e_{ij})}$$

where $S_{i}$ and $S_{j}$ are the sizes of the largest fully connected subgraphs containing node i and node j, respectively, and $S(e_{ij})$ is the size of the largest fully connected subgraph containing edge $e_{ij}$.

#### 2.1.4. Diffusion Intensity (DI)

Diffusion Intensity (DI) [11] evaluates the significance of an edge based on the properties of the neighboring nodes of the two connected nodes. It is defined as

(4) $$DI(e_{ij})=\frac{{\;n_{i\;ex\;j}+n}_{j\;ex\;i\;}}{2}$$

where $n_{i\;ex\;j\;}$ is the number of neighbor nodes of node i(excluding node j) that are not connected to node j, and $n_{j\;ex\;i\;}$ is the number of neighbor nodes of node j (excluding node i) that are not connected to node i.

#### 2.1.5. Edge Importance Metric (EI)

The edge importance metric (EI) [22] method takes into account the impact of triangles, diagonal quadrilaterals, fully connected quadrilaterals, and empty quadrilaterals on the network topology and propagation dynamics. It also considers the influence of edges on the global network characteristics (the average path length). The formula is as follows:

(5) $$EI(e_{ij})=\frac{N_{∆}^{\prime }+N_{\alpha }^{\prime }+N_{\eta }^{\prime }+N_{\beta }^{\prime }+N_{∎}^{\prime }}{N_{∆}+N_{\alpha }+N_{\eta }+N_{\beta }+N_{∎}}\times |L-L^{\prime }|$$

$N_{∆}^{\prime }\;and\;N_{\alpha }^{\prime }$ represent the actual number of triangles and empty quadrilaterals, respectively, considering edge $e_{ij}$ as an outer edge. $N_{\eta }^{\prime }$ indicates the actual count of diagonal quadrilaterals in the first-order neighborhood, $N_{\beta }^{\prime }$ refers to the count of diagonal quadrilaterals in the second-order neighborhood with edge $e_{ij}$ as an inner edge, and $N_{∎}^{\prime }$ signifies the actual count of fully connected quadrilaterals. $N_{∆}$ is the maximum number of triangles with edge $e_{ij}$ as an outer edge, while $N_{\alpha }^{\prime }$ and $N_{\eta }^{\prime }$ denote the maximum possible counts of empty quadrilaterals and diagonal quadrilaterals, respectively. $N_{\beta }^{\prime }$ indicates the maximum number of diagonal quadrilaterals with edge $e_{ij}$ as an inner edge, and $N_{∎}^{\prime }$ represents the maximum possible count of fully connected quadrilaterals. Finally, L is the average path length of the original network, and $L^{\prime }$ is the average path length after contracting nodes *i* and *j* along edge $e_{ij}$.

#### 2.1.6. Second-Order Neighborhood (SN)

The second-order neighborhood (SN) [14] aims to identify potential bridges between communities. In a network, if node i and node j do not share common neighbors, the edge between nodes i and node j is likely to be a potential bridge between two different communities. Therefore, the second-order neighborhood index is defined as

(6) $$SN(e_{ij})=\frac{\left|{NN}_{i/j}⋂{NN}_{j/i}\right|}{\left|{NN}_{i/j}⋃{NN}_{j/i}\right|}$$

where ${NN}_{i/j}$ represents the set of nodes that can be reached from node i via a path with a length of 2 without passing through node j, and ${NN}_{j/i}$ represents the set of nodes that can be reached from node j via a path with a length of 2 without passing through node i. The smaller the second-order neighborhood index of an edge, the more likely it is to be a bridge connecting two communities, and thus the more important it is within the network.

### 2.2. Proposed Methods

#### 2.2.1. The Enhanced Neighborhood (EN) Method

Among critical edge identification methods based on local information, the second-order neighborhood (SN) method has demonstrated good performance in identifying critical edges. However, the SN method only considers local information, limiting its ability to comprehensively assess edge importance. In sparse networks, its performance degrades due to the absence of second-order neighbors for many nodes, resulting in an SN value of zero for numerous edges.

**Figure 1:**
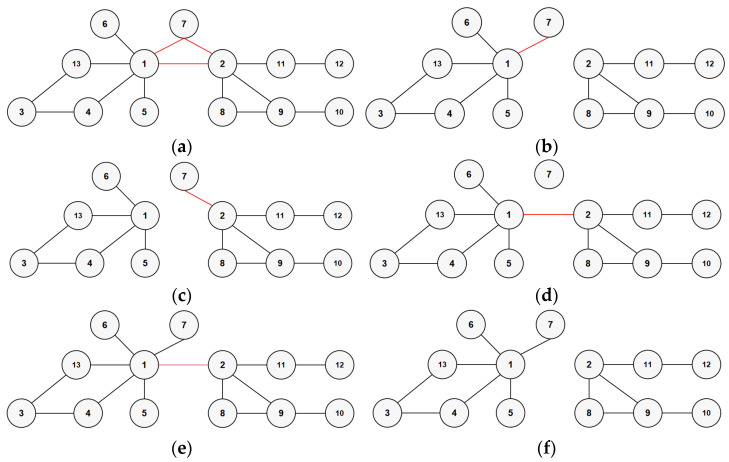
(**a**–**d**) show the original network; the network after removing edges (1, 2) and (2, 7); the network after removing edges (1, 2) and (1, 7); and the network after removing edges (2, 7) and (1, 7), respectively. (**e**) presents a network without triangular structures, and (**f**) shows the network after removing edge (1, 2) from the network in (**e**). In these figures, important edges are marked with red lines, and the others are black lines.

As shown in Figure 1a, in a connected sparse network, practical experience indicates that the three red edges, including (1, 2), (1, 7), and (2, 7), are relatively important in the sparse network. In particular, edge (1, 2) is the most critical, as removing any two of the three edges, including (1, 2), would split the connected simple network into two significantly larger connected components, as illustrated in Figure 1b, where edges (1, 2) and (2, 7) are removed, and in Figure 1c, where edges (1, 2) and (1, 7) are removed.

However, the edge importance ranking obtained by the SN method, as shown in Table 1, reveals a significant oversight: SN only correctly identifies the importance of edges (1, 7) and (2, 7) while failing to recognize the most critical edge, (1, 2). This is because there are no common second-order neighbors between node 1 and node 2. This is a considerable shortcoming, as even after removing the two edges deemed important by SN, the connectivity of the graph remains largely unchanged, as illustrated in Figure 1d.

To address this issue, this paper proposes an enhanced neighborhood (EN) method that introduces the first-order neighborhood (FN) based on the second-order neighborhood (SN). This approach aims to improve the accuracy of edge importance assessments by integrating more local information. As a supplement to local information, FN helps identify the importance of critical edges by evaluating the direct neighbor relationships between two nodes. The formula for FN is as follows:

(7) $$FN(e_{ij})=\frac{\left|N_{i/j}⋂N_{j/i}\right|}{\left|N_{i/j}⋃N_{j/i}\right|}$$

By considering first-order neighborhood, we can obtain more information about the node connectivity, thereby addressing the limitations of the second-order neighborhood method in sparse networks. The formula for the enhanced neighborhood (EN) method is as follows:

(8) $$EN(e_{ij})=\frac{2\times |NN_{i/j}⋂{NN}_{j/i}|}{\left|NN_{i/j}⋃{NN}_{j/i}\right|}+\frac{3\times |N_{i/j}⋂N_{j/i}|}{\left|N_{i/j}⋃N_{j/i}\right|}$$

It can be simplified into the following:

(9) $$EN(e_{ij})=2\;SN(e_{ij})+3FN(e_{ij})$$

where the coefficient is derived from experiments. As shown in Table 1, the EN method can identify the importance of edges (1, 7), (2, 7), and (1, 2). Therefore, EN is more accurate than the SN method in identifying critical edges. By introducing FN, EN is enabled to identify important edges such as (1, 2), thereby resolving the problem of overlooking certain critical edges due to the SN method.

#### 2.2.2. Global–Local Hybrid Centrality (GLHC)

The enhanced neighborhood method (EN) proposed in Section 2.2.1, while correctly identifying three important edges, still faces several issues, as shown in Table 1:

(1) Inaccurate ranking: Although the EN method can correctly identify the top three edges, the ranking is not precise, with the most important edge (1, 2) being misclassified as the third most important.
(2) The inability to identify edges outside of triangular structures results in most edges scoring zero: except for the top three ranked edges, all the other edges receive a score of zero because there are no triangular structures present, which means the nodes connected by these edges lack common second-order or first-order neighbors.

The issue mentioned in point (2) is very critical. For edges that do not form triangular structures, the EN method is unable to effectively assess their importance due to the lack of common first-order and second-order neighbors between the two connected nodes. As a result, in complex networks with inter-community structures, while this method can accurately identify key edges within communities, it often overlooks these crucial bridging edges that connect different communities due to the absence of common neighbor relationships.

For instance, in the network shown in Figure 1e, the most critical edge is (1, 2) because removing edge (1, 2) can split the network into two larger connected subnetworks, which cannot be achieved by removing any other edge, as shown in Figure 1f. However, the EN method assigns an importance score of 0 to all edges because there are no triangular structures present, meaning the nodes connected by these edges do not share common second-order or first-order neighbors. As indicated in Table 2, both the SN method and the EN method assign an importance score of 0 to all edges.

However, we can see from Table 1 that using the edge betweenness (EB) method, which is based on global information, although it successfully identifies the importance of edge (1, 2), edges (1, 7) and (2, 7) are ranked last. Although these edges also bridge edges between communities, the EB method’s definition of edge betweenness relies on the shortest path information for the overall network. As a result, it lacks local information to assist in its judgment, which may lead to an inaccurate reflection of the importance of certain bridging edges between communities when handling specific network structures.

To address these issues, this paper proposes the Global–Local Hybrid Centrality (GLHC) method, which combines the local EN method with the global EB method. This approach is not only sensitive to local structures within communities but also capable of identifying bridging edges that span across communities. The formula is as follows:

(10) $$\begin{array}{c} GLHC(e_{ij})=3\;EB(e_{ij})+\frac{1}{2}\;EN(e_{ij})\\ =3\;EB(e_{ij})+1\;SN(e_{ij})+\frac{3}{2}\;FN(e_{ij}) \end{array}$$

The average time complexity of EB is *O(MN)*, while the average time complexity of FN and SN is *O(MK)*, where M denotes the number of edges, N represents the number of nodes, and K signifies the average degree. Therefore, the time complexity of GLHC is primarily dominated by the complexity of EB, expressed as *O(MN)*.

**Figure 2:**
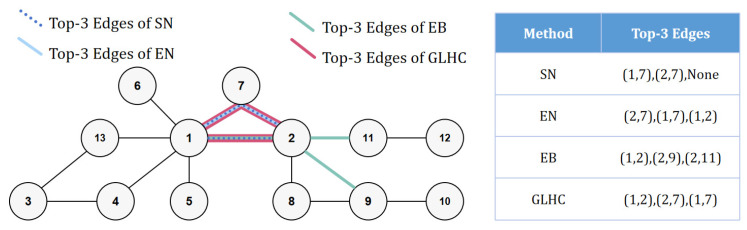
The top three important edges determined by the SN, EN, EB, and GLHC methods, respectively.

The results of critical edge identification using GLHC in the network in Figure 1a are shown in Table 1, demonstrating that this method successfully addresses all the issues:

(1) Accurate identification and ranking: As shown in Figure 2, the GLHC method is the only one that not only accurately identifies the top three important edges but also correctly ranks them, with edge (1, 2) being correctly identified as the most important and ranked first.
(2) Capable of identifying edges that are not part of triangular structures, and all edges receive scores: Even if certain edges lack common first-order or second-order neighbors, GLHC still generates reasonable scores and corresponding rankings for them, unlike the EN method, which simply assigns a score of zero.
(3) Compensates for the lack of local information in global methods: Compared to EB, the GLHC method is able to accurately identify the importance of edges (1, 7) and (2, 7).

Furthermore, for the network in Figure 1e, as shown in Table 2, GLHC can correctly identify the most important edge (1, 2) because it incorporates EB, even though it is not part of a triangular structure. For edges that do not constitute triangular structures, GLHC can still assign importance scores.

In summary, by combining local information and global information, the GLHC method provides a more comprehensive assessment of edge importance, specifically reflected in the following:

(1) Local information aids in capturing fine-grained structures within communities: Within the network structure of communities, the enhanced neighborhood coefficient method effectively identifies close connections between nodes. In complex networks, communities often exhibit high levels of clustering, and local information among nodes can reveal their core roles within the community. Therefore, the GLHC method leverages local neighbor information to more accurately assess the importance of edges within communities, avoiding oversight of critical internal structures.
(2) Global information provides a macro perspective across communities: By incorporating global information, the GLHC method can identify bridging edges that connect different communities. These bridging edges are often the primary channels for inter-community information transfer and resource flow, which are crucial to the overall connectivity and dissemination of information within the network. Neglecting these edges can lead to significant biases in assessing network importance, while the GLHC method ensures that these critical bridging edges are not overlooked by integrating global information.

The combination of both local and global information enables the GLHC method to evaluate edge importance on multiple levels, achieving a more comprehensive and precise identification of key edges.

## 3. Results and Discussion

### 3.1. Datasets

In this study, we evaluated the performance of GLHC on twelve empirical unweighted networks from different domains, along with three synthetic networks. These networks were as follows: (1) RhesusBrain [23]: A connectivity network of the rhesus monkey brain; (2) IndustryPartner [23]: A collaboration network of partners in the internet industry; (3) InfectHyper [23]: A dataset specifically used to study infection spreading and diffusion processes in infection networks; (4) Ego [24] 1 to 3: Social networks of three specific Facebook users; (5) RoadMinnesota [23]: A road transportation network of Minnesota; (6) Drosophila [25]: A dataset of the Drosophila brain network, in which edges represent the fiber tracts connecting various brain regions, used to study the brain′s structure and connectivity properties; (7) Yeast [26]: Protein–protein interaction network for yeast; (8) GR-QC [27]: An author collaboration network of electronic publications on Arxiv in the General Relativity and Quantum Cosmology category; (9) Oregon [28]: A network topology dataset representing connections between Internet Autonomous Systems; (10) Company [29]: A social network of blue-verified users related to companies, brands, and organizations on Facebook in the United States. Table 3 lists the characteristics of these networks; (11) ER (Erdős–Rényi): A random graph model where each edge is included in the graph with a probability p, producing a network characterized by a Poisson degree distribution; (12) BA (Barabási–Albert): A scale-free network model that generates networks using a preferential attachment mechanism, leading to a power-law degree distribution; (13) WS (Watts–Strogatz): A small-world network model that creates networks with a high clustering coefficient and a short average path length by randomly rewiring edges in a regular ring lattice.

The parameters for the three generated synthetic networks are as follows: for the ER network, the number of nodes is set to 20,000 with an edge generation probability of 0.01; for the BA network, the number of nodes is set to 20,000, with each new node connecting to 3 existing nodes; and for the WS network, the number of nodes is set to 5000, with each node having 6 neighbors and a rewiring probability of 0.1.

### 3.2. Robustness

Network robustness [30] refers to a network’s ability to maintain its functionality and performance despite various failures, attacks, or unexpected disruptions. The robustness metric id defined as follows:

(11) $$R=\frac{1}{E}\sum_{l=1}^{E}\xi_{l}$$

where E represents the total number of edges in the original network, l represents the index of the edge to be removed, and the order of edge removal is determined by the edge importance method. $\xi_{l}$ denotes the proportion of nodes in the largest connected component after the removal of the edge indexed by l, relative to the total number of nodes in the original network. This proportion reflects the network’s connectivity status after edge removal. $\frac{1}{E}$ is a normalization factor ensuring that the robustness value remains between 0 and 1, enabling a fair comparison of robustness across networks with varying numbers of edges.

The robustness metrics are actually indicators that measure the degree of network disintegration during the optimal percolation process. It assesses network vulnerability by quantifying its ability to retain connectivity after the removal of critical edges. A lower robustness value indicates that the proportion of nodes in the largest connected component is smaller after the removal of critical edges, signifying that network connectivity has been more severely affected during the edge percolation process. Hence, lower robustness suggests higher accuracy in critical edge identification methods, as it identifies and removes the edges that most affect the network’s connectivity better.

We compared the performance of the GLHC method against six baseline methods—EB, DP, DI, BN, EI, and SN—and the EN method proposed in Section 2.2.1 across fifteen networks using robustness metrics. For each method, we ranked the importance of the edges in the network and removed them in descending order of importance until no edges remained, calculating the robustness metric for each method. Table 4 presents the robustness performance of each critical edge identification method across different networks, with the method achieving the lowest robustness in each network highlighted in bold.

A lower robustness metric means that removing edges based on the rankings provided by the critical edge identification method causes the network to disintegrate more rapidly. The results indicate that SN performs best among the six existing benchmark methods on most datasets. Meanwhile, the global methods EB and BN exhibit a subpar performance on the majority of datasets. However, in the RhesusBrain and RoadMinnesota datasets, which contain a significant number of inter-community edges, the global information-based methods, EB and BN, outperform SN.

EN introduces FN on top of SN, and it can be observed that EN achieves an improvement over SN on several datasets. The GLHC method further integrates both local and global information approaches, achieving the lowest robustness scores across all twelve datasets and demonstrating a superior overall performance.

### 3.3. The Effect of Connectivity Degradation

The evaluation of edge importance methods often involves metrics related to the connectivity degradation effect, as the importance of an edge is typically associated with its contribution to the network connectivity. If removing specific edges rapidly decreases the network connectivity, this suggests that those edges are crucial for maintaining the network structure. The connectivity degradation index [31] is defined as follows:

(12) $$k=\frac{N_{After}}{N}$$

where $N_{After}$ represents the number of nodes in the largest connected component after the removal of an edge, and N is a normalization parameter representing the number of nodes in the largest connected component of the original network, ensuring this metric is applicable to networks of different scales.

We compared the connectivity degradation index of GLHC with that of baseline methods using edge percolation dynamics. This involves sequentially removing the most critical edges identified by each method and calculating the connectivity degradation index at each step until all edges have been removed. If connectivity degradation occurs more rapidly during the edge percolation process, this indicates the higher accuracy of the method. In fact, the connectivity decline metric mentioned in this section is closely related to the robustness metric in Section 3.2, as both are used to measure the extent of network disintegration and collapse during the optimal percolation process. The robustness metric essentially measures the area enclosed by the connectivity decline curve and the line segments x = 0 and x = 0 during the process of edge removal. The size of this area is inversely related to the performance of the critical edge identification method; a smaller area indicates superior performance in identifying critical edges, and vice versa. Generally, a more robust critical edge identification method will also demonstrate a better overall performance in terms of connectivity decline. It is important to note that while the connectivity decline metric and the robustness metric are related, they focus on different aspects. The advantage of the connectivity decline metric lies in its ability to fully reflect the changes in network connectivity during the edge removal process, providing a clear view of the entire process. However, its disadvantage is the lack of a quantifiable assessment, making it difficult to evaluate the network’s robustness using specific numerical values. In contrast, the robustness metric provides a clear numerical value for assessing the effectiveness of critical edge identification methods but may not comprehensively capture the entire process of connectivity changes. Therefore, using both metrics can complement each other, offering an intuitive view of the process while enabling in-depth evaluation and optimization of the performance of critical edge identification methods through quantifiable results.

**Figure 3:**
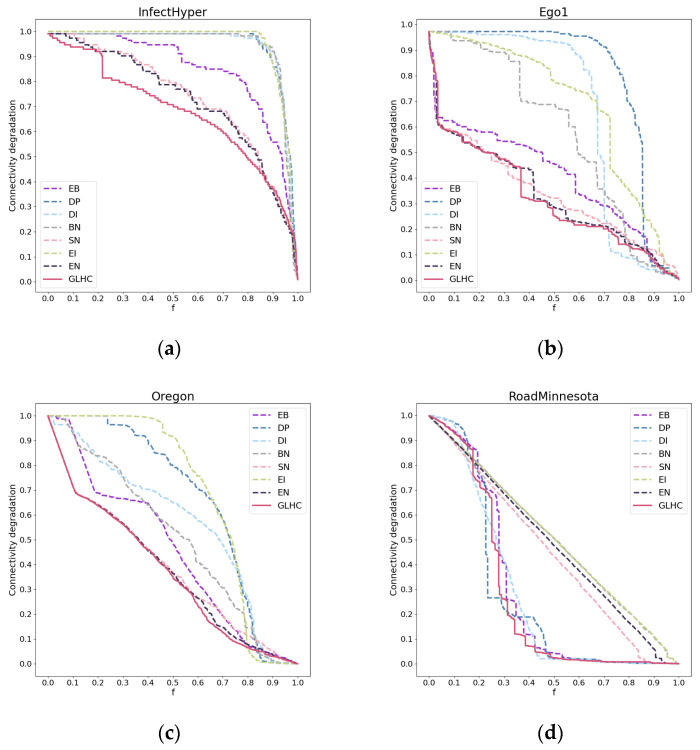
Subfigures (**a**–**d**) illustrate the performance in terms of accuracy of GLHC in the InfectHyper, Ego1, Oregon, and RoadMinnesota networks. The horizontal axis represents the proportion of nodes removed, and the vertical axis represents the ratio of the number of nodes in the largest remaining connected subgraph after node removal to the number of nodes in the original network.

The performance in terms of accuracy in the InfectHyper, Ego1, Oregon and RoadMinnesota networks is shown in Figure 3, while the performance across all networks is presented in Appendix A.

The experimental results show that in the InfectHyper, Ego1, and Oregon datasets, the SN method causes a faster decline in connectivity compared to the other benchmark methods (EB, DP, DI, BN, and EI), indicating that SN performs better in identifying critical edges. This is also the reason why the GLHC method is designed as an improvement based on SN. The EN method proposed in this paper introduces FN based on SN, addressing the issue of reduced accuracy in sparse networks due to the lack of common second-order neighbors between many nodes, making EN perform better than SN in sparse networks (i.e., the latter segment of the *x* axis). However, on the RoadMinnesota dataset, SN does not perform well due to the presence of numerous inter-community bridging edges that do not form triangles, which SN fails to effectively identify. Meanwhile, the EN method, despite incorporating additional neighbor information, also neglects the significance of bridging edges, resulting in inferior performance relative to SN. In contrast, the EB method, which relies on global information, excels in this type of network topology.

The GLHC method combines SN, FN, and EB, allowing it to capture both local and global information. Whether it is the intra-community structure that is more suited to local information or the inter-community structure that is more suited to global information, the GLHC method adapts well to both. As a result, it performs excellently across all fifteen networks (as shown in Figure 2 and Appendix A), enabling the network connectivity to decline at the fastest rate and achieving the highest accuracy in identifying critical edges.

### 3.4. Monotonicity

A reliable edge importance ranking method should uniquely determine the importance of each edge, rather than broadly categorizing them. This paper uses the monotonicity indicator to measure the precision of different edge importance ranking methods. The monotonicity [32] of the ranking is defined as follows:

(13) $$M={\left[1-\frac{\sum_{i\in L}b_{i}(b_{i}-1)}{\left|E|(\left|E\right|-1\right)}\right]}^{2}$$

where |E| represents the number of edges in the network, *L* is the ranking list formed by the node importance ranking method, and $b_{i}$ represents the number of edges ranked at position i in the list. The value of *M* ranges from 0 to 1, with values closer to 1 indicating stronger monotonicity, meaning better consistency in the ranking of edge importance.

Table 5 presents the results for the monotonicity of the various edge importance methods across different networks, with the method ranked first in each network highlighted in bold. We can observe that across fifteen networks, GLHC demonstrates the strongest monotonicity, followed by edge betweenness. This indicates that the GLHC method more accurately reflects the importance of edges in the ranking process and maintains a higher ranking consistency, thereby offering greater reliability in practical applications.

## 4. Conclusions

This paper proposes a novel metric for assessing edge importance in networks, termed Global–Local Hybrid Centrality (GLHC). The GLHC method integrates three key indicators—second-order neighborhood, first-order neighborhood, and edge betweenness—to comprehensively assess the edge importance in a network. The experimental results demonstrate that by considering both the global and local roles of edges within the network structure, GLHC can more accurately identify critical edges essential for network stability and connectivity.

Overall, the GLHC method demonstrates high accuracy and reliability in identifying key edges, providing an effective approach for the analysis and optimization of complex networks. Its comprehensive performance and robustness offer reliable support for network systems across various application scenarios, including traffic planning, power grid optimization, social network analysis, biological network research, and financial risk management. Identifying and protecting critical edges can significantly enhance the stability and robustness of these systems, optimizing resource utilization and information transmission, thereby providing crucial support for societal development and economic growth.

However, the current GLHC method’s formula structure and weights were derived through reverse engineering of the experimental results. While this approach has proven effective, it has certain limitations. Future research could explore the relationships between network structure, properties, and the number of node neighbors to systematically determine both the formula structure and the corresponding weights. In addition, while the GLHC method improves the accuracy of critical edge identification by incorporating global algorithms, it also increases the computational time complexity. As a result, the GLHC method is more suitable for medium- and small-scale networks, particularly in scenarios where high precision in identifying critical edges is required. For example, in fault analysis and the optimization of power grids, accurately identifying critical edges is essential for ensuring grid stability. In the case of a regional power grid, although the network is relatively medium to small in scale, misidentifying critical edges could lead to severe disruptions in power transmission, potentially causing large-scale blackouts. In such a scenario, the high-precision identification capability of the GLHC method becomes especially important. As research progresses, future efforts could focus on optimizing the algorithm to reduce the time complexity and further enhance the applicability of the GLHC method.

The application of the GLHC method is limited to undirected static graphs. Future research can consider the impact of edge directionality on the network structure further and adjust the calculation of related metrics in directed graphs. Additionally, time windows or time-weighting mechanisms could be introduced to analyze the dynamic changes in edges at different time points, thus extending the GLHC method to dynamic graphs, thereby enhancing its applicability in more complex network environments.

Additionally, the GLHC method holds potential for diffusion source inference [33,34], as identifying critical edges in a network can help locate the origin of diffusion. In incomplete networks, this method can also be applied to containing diffusion and influence maximization [35,36,37]. By identifying and controlling critical edges, it can effectively stop diffusion or enhance the spread of information. Future research could explore the performance of the GLHC method in these applications, thereby further expanding its applicability to real-world problems.

## Figures and Tables

**Table 1:** The results of critical edge identification using the Second-Order Neighborhood (SN), The Enhanced Neighborhood (EN), Edge Betweenness (EB), and Global–Local Hybrid Centrality (GLHC) methods on the original network shown in Figure 1a, with scores rounded to two decimal places and edges (1, 2), (1, 7), and (2, 7) highlighted in bold.

	SN		EN		EB		GLHC	
Rank	Edge	Score	Edge	Score	Edge	Score	Edge	Score
1	**(1, 7)**	**0.5**	**(2, 7)**	**1.75**	**(1, 2)**	**0.46**	**(1, 2)**	**1.57**
2	**(2, 7)**	**0.5**	**(1, 7)**	**1.6**	(2, 9)	0.28	**(2, 7)**	**1.10**
3	(1, 4)	0.0	**(1, 2)**	**0.38**	(2, 11)	0.28	**(1, 7)**	**1.03**
4	(1, 5)	0.0	(1, 4)	0.0	(1, 4)	0.20	(2, 9)	0.85
5	(1, 6)	0.0	(1, 5)	0.0	(1, 13)	0.20	(2, 11)	0.85
6	(1, 13)	0.0	(1, 6)	0.0	(1, 5)	0.15	(1, 4)	0.60
7	**(1, 2)**	**0.0**	(1, 13)	0.0	(1, 6)	0.15	(1, 13)	0.59
8	(2, 8)	0.0	(2, 8)	0.0	(2, 8)	0.15	(1, 5)	0.46
9	(2, 9)	0.0	(2, 9)	0.0	(9, 10)	0.15	(1, 6)	0.46
10	(2, 11)	0.0	(2, 11)	0.0	(11, 12)	0.15	(2, 8)	0.46
11	(4, 3)	0.0	(4, 3)	0.0	(4, 3)	0.08	(9, 10)	0.46
12	(13, 3)	0.0	(13, 3)	0.0	(13, 3)	0.08	(11, 12)	0.46
13	(9, 10)	0.0	(9, 10)	0.0	**(1, 7)**	**0.08**	(4, 3)	0.25
14	(11, 12)	0.0	(11, 12)	0.0	**(2, 7)**	**0.08**	(13, 3)	0.25

**Table 2:** The results of critical edge identification using the SN, EN, EB, and GLHC methods in the network shown in Figure 1e, with scores rounded to two decimal places and edge (1, 2) highlighted in bold.

	SN		EN		EB		GLHC	
Rank	Edge	Score	Edge	Score	Edge	Score	Edge	Score
1	(1, 7)	0.0	(1, 7)	0.0	**(1, 2)**	**0.54**	**(1, 2)**	**1.62**
2	(11, 12)	0.0	(11, 12)	0.0	(2, 9)	0.28	(2, 9)	0.85
3	(1, 4)	0.0	(1, 4)	0.0	(2, 11)	0.28	(2, 11)	0.85
4	(1, 5)	0.0	(1, 5)	0.0	(1, 4)	0.20	(1, 4)	0.60
5	(1, 6)	0.0	(1, 6)	0.0	(1, 13)	0.20	(1, 13)	0.60
6	(1, 13)	0.0	(1, 13)	0.0	(1, 5)	0.15	(1, 5)	0.46
7	**(1, 2)**	**0.0**	**(1, 2)**	**0.0**	(1, 6)	0.15	(1, 6)	0.46
8	(2, 8)	0.0	(2, 8)	0.0	(1, 7)	0.15	(1, 7)	0.46
9	(2, 9)	0.0	(2, 9)	0.0	(2, 8)	0.15	(2, 8)	0.46
10	(2, 11)	0.0	(2, 11)	0.0	(9, 10)	0.15	(9, 10)	0.46
11	(4, 3)	0.0	(4, 3)	0.0	(11, 12)	0.15	(11, 12)	0.46
12	(13, 3)	0.0	(13, 3)	0.0	(4, 3)	0.08	(4, 3)	0.25
13	(9, 10)	0.0	(9, 10)	0.0	(13, 3)	0.08	(13, 3)	0.25

**Table 3:** Basic topological features of network data. The Networks column presents the names of the networks. E and N represent the number of nodes and edges, respectively. <k> represents the average degree of all nodes in the network. C represents the clustering coefficient, describing the degree to which a node’s neighbors are interconnected. r represents the assortativity coefficient, measuring the tendency of nodes to connect with other nodes of a similar degree within the network.

Networks	E	N	<k>	C	r
RhesusBrain	582	91	12.7912	0.8601	−0.7698
IndustryPartner	630	219	5.7534	0.1762	−0.2168
InfectHyper	2196	113	38.8673	0.5348	−0.1226
Ego1	2519	333	15.1291	0.5082	0.2360
Ego2	3192	224	28.5	0.5443	0.2227
Ego3	4813	534	18.02621	0.5437	0.2224
RoadMinnesota	3303	2642	2.5004	0.0159	−0.1848
Drosophila	9016	1781	10.1246	0.2628	−0.0943
Yeast	7182	2361	6.0838	0.1301	−0.0845
GR-QC	14,496	5242	5.5307	0.5296	0.6591
Oregon	31,180	10,900	5.7211	0.3525	−0.1556
Company	52,310	14,113	7.4130	0.2392	0.0129
ER	59,991	20,000	5.9991	0.0025	−0.0308
BA	60,000	20,000	6.0000	0.4412	−0.0154
WS	124,579	5000	49.8316	0.0100	−0.0008

**Table 4:** The comparison of robustness across different critical edge identification measures in various networks, with the method with the lowest robustness in each network highlighted in bold.

Networks	EB	DP	DI	BN	EI	SN	EN	GLHC
RhesusBrain	0.5966	0.8389	0.6882	0.5432	0.7861	0.6687	0.5796	**0.5320**
IndustryPartner	0.5673	0.7009	0.6830	0.6046	0.6792	0.5319	0.5115	**0.4711**
InfectHyper	0.8488	0.9525	0.9430	0.9419	0.9489	0.7279	0.7149	**0.6785**
Ego1	0.4440	0.8328	0.6941	0.6127	0.6839	0.3687	0.3571	**0.3463**
Ego2	0.5930	0.9049	0.8577	0.8113	0.6907	0.5630	0.5523	**0.5374**
Ego3	0.3233	0.8418	0.6712	0.5803	0.7087	0.3586	0.3318	**0.2983**
RoadMinnesota	0.2851	0.2640	0.2737	0.5030	0.5038	0.4441	0.4805	**0.2625**
Drosophila	0.6440	0.8598	0.8248	0.7614	0.7226	0.6096	0.6013	**0.5826**
Yeast	0.5295	0.6995	0.6234	0.5727	0.5817	0.4220	0.4198	**0.4147**
GR-QC	0.2406	0.4533	0.2623	0.1963	0.3446	0.2189	0.2035	**0.1926**
Oregon	0.5445	0.7245	0.6211	0.5615	0.7260	0.4737	0.4912	**0.4434**
Company	0.5317	0.7591	0.6577	0.5617	0.5852	0.4065	0.4289	**0.4001**
ER	0.8152	0.8386	0.8217	0.8424	0.8341	0.7539	0.7467	**0.7057**
BA	0.7291	0.7304	0.7261	0.7369	0.7599	0.6978	0.6818	**0.6338**
WS	0.2868	0.6227	0.2476	0.5880	0.6085	0.2032	0.2065	**0.1930**

**Table 5:** Comparison of the monotonicity of various edge importance methods across different networks. The method ranked first in terms of monotonicity for each network is highlighted in bold (including cases where there are ties for first place).

Networks	EB	DP	DI	BN	EI	SN	EN	GLHC
RhesusBrain	0.9945	0.9637	0.9374	0.8371	0.9673	0.8982	0.9749	**0.9951**
IndustryPartner	0.9855	0.9849	0.9849	0.7621	0.9838	0.9826	0.9855	**0.9922**
InfectHyper	0.8487	0.9525	0.9429	0.9419	0.9488	0.7279	0.9048	**0.9685**
Ego1	0.9996	0.9964	0.9712	0.9701	0.9876	0.9983	0.9991	**0.9997**
Ego2	**0.9999**	0.9981	0.9684	0.9885	0.9813	0.9978	**0.9999**	**0.9999**
Ego3	0.9998	0.9964	0.9700	0.9774	0.9761	0.9981	0.9998	**0.9999**
RoadMinnesota	**0.9984**	0.6194	0.4970	0.5138	0.8454	0.8581	0.8984	**0.9984**
Drosophila	**0.9997**	0.9969	0.9856	0.9502	0.9699	0.9996	0.9957	**0.9997**
Yeast	0.9862	**0.9910**	0.9554	0.7925	0.9689	0.9829	0.9472	0.9830
GR-QC	0.9083	0.9807	0.8684	0.4174	0.9347	0.9813	0.9770	**0.9920**
Oregon	**0.9994**	0.9914	0.9882	0.9362	0.9876	0.9993	0.9313	**0.9994**
Company	**0.9998**	0.9923	0.9695	0.8555	0.9867	0.9924	0.9780	0.9934
ER	**1.0**	0.9668	0.8796	0.9839	0.7367	0.9378	0.9412	**1.0**
BA	**0.9999**	0.9692	0.9398	0.9818	0.7076	0.9821	0.9846	**0.9999**
WS	0.9997	0.6095	0.7756	0.6759	0.9726	0.9126	0.9373	**0.9999**

## Data Availability

The data are contained within the article.

## Appendix A

The performance of GLHC and the baseline methods on 12 networks.
